# FPGA-Based Smart Sensor to Detect Current Transformer Saturation during Inrush Current Measurement

**DOI:** 10.3390/s23020744

**Published:** 2023-01-09

**Authors:** G. de J. Martínez-Figueroa, Felipe Córcoles-López, Santiago Bogarra

**Affiliations:** Department of Electrical Engineering, ESEIAAT, Universitat Politècnica de Catalunya, 08222 Terrassa, Spain

**Keywords:** current transformer, saturation, smart sensor, FPGA, inrush current

## Abstract

Current transformer saturation affects measurement accuracy and, consequently, protection reliability. One important concern in the case of overcurrent protections is the discrimination between faults and inrush current in power transformers. This paper presents an FPGA-based smart sensor to detect current transformer saturation, especially during inrush current conditions. Several methods have been proposed in the literature, but some are unsuitable for inrush currents due to their particular waveform. The proposed algorithm implemented on the smart sensor uses two time-domain features of the measured secondary current: the second-order difference function and the third-order statistic central moment. The proposed smart sensor presents high effectiveness and immunity against noise with accurate results in different conditions: different residual flux, resistive burdens, sampling frequency, and noise levels. The points at which saturation starts are detected with an accuracy of approximately 100%. Regarding the end of saturation, the proposed method detects the right ending points with a maximum error of a sample. The smart sensor has been tested on experimental online and real-time conditions (including an anti-aliasing filter) with accurate results. Unlike most existing methods, the proposed smart sensor operates efficiently during inrush conditions. The smart sensor presents high-speed processing despite its simplicity and low computational cost.

## 1. Introduction

Current transformers (CTs) are essential instrumentation elements between power systems and protective relays or measurement devices. The main purpose of a CT is to reduce a large primary current to a smaller secondary level suitable for connected devices. The accurate reproduction of the CT primary current is a relevant concern. When a CT saturates (due to core nonlinearity), it provides distorted signals that reduce the measurement accuracy and, consequently, protection reliability [1]. The saturation occurs, mainly, in two scenarios [2]: (1) when the symmetrical primary current is too large and (2) when there is a transient current with a significant direct component (DC), as happens in fault currents or inrush currents due to power transformer energization. One of the main concerns in the case of overcurrent protections is the discrimination between faults and inrush currents in power transformers. Thus, the distorted CT secondary current must be compensated to mitigate the protective relays’ vulnerability to CT saturation and ensure their operation.

There are several methodologies to recover the primary current from distorted CT secondary current [3,4,5,6,7,8,9,10,11]. These methodologies require accurate knowledge of the zones where the current is distorted. Therefore, detecting the saturation intervals in the measured current is an essential first step to overcoming the problem. The most classical method for this purpose is presented in [3], which detects CT saturation by comparing the measured CT secondary current with the theoretical primary current. The last one is obtained using the previously estimated CT saturation curve and the CT model. This method may be inadequate to detect CT saturation in real-time conditions.

In [12], an algorithm is presented to detect CT saturation based on locating the inflection points where saturation begins and ends, by using the third difference of the current and a preset threshold. The algorithm has also been implemented in a digital signal processor, with good results in detecting saturation of fault currents, regardless of the CT core residual flux levels. The methodology presented in [13] is also based on detecting the abrupt change of the current waveform when saturation occurs, using a combination between the second-order derivative and zero crossing techniques. The second derivative technique uses an adaptive threshold obtained by curve fitting using unsaturated samples from the secondary CT current, improving the results of [12].

Using the shape differences between the distorted and undistorted sections of CT secondary current, [14] proposes a method to detect CT saturation quickly. First, a symmetrical variable-length window is defined for the secondary CT current. The least square error technique is employed to process the current samples inside this window and to make the estimation of the current samples before and after the window. CT saturation can be detected based on the difference between these two estimations.

Other methodologies with similar approaches, based on detecting the abrupt changes of the saturated current waveform, have been proposed in [15,16,17]. However, these methodologies can lead to false CT saturation detections in inrush current occurrence because of the abrupt changes at each period due to the power transformer saturation, even without CT saturation.

Recent algorithms with different approaches have been developed [11,18,19,20,21], but there remains a lack of algorithms designed for inrush conditions.

A hybrid method based on a physical and a data-driven model is proposed in [11] to detect CT saturation. The essential idea is to use the data-driven model (a Fully Convolutional Network) to handle the complex and nonlinear characteristics of the CT and to adopt a physical model, i.e., a short-circuit current model, to reproduce the true short-circuit current waveform. Hence, the combination of the data and physical models is implemented in sequence. This method makes next assumptions: the CT magnetizing inductance is constant and the residual flux is null. Another artificial neural network (ANN) based CT saturation detector is presented in [18]. A genetic algorithm scheme is used for ANN optimization.

In [19], the author proposes a CT saturation detection algorithm for bus-bar differential protection based on the measurement of the power system source impedance seen at the relay location. Impedance calculation is based on a first-order differential equation of an RL model of a transmission line, with three consecutive samples of bus-bar voltage and CT secondary current.

A Savitzky–Golay filter is used in [20] to detect CT saturation. This filter is a method of smoothing and differentiating noisy discrete data based on local least square polynomial approximation. In [21], the authors propose an algorithm that combines the Savitzky–Golay filter with the Empirical Mode Decomposition technique, which decomposes a signal into its physically meaningful components.

Other methodologies are based on the frequency spectrum of saturated current [22,23]. These methodologies use time-frequency techniques such as Wavelet transform, and Hilbert-Huang transform. This approach is not useful with inrush currents due to their high harmonic content.

This work proposes the design of an FPGA-based smart sensor to detect CT saturation through the combined use of two simple time-domain features of the measured secondary current: the second-order difference function and the third-order statistic central moment. A smart sensor is useful monitoring system with high-performance instrumentation and online signal processing for true real-time monitoring [24,25]. A smart sensor must include at least a primary sensor, integrated signal processing capabilities, and communication, but it can also incorporate data logging, learning, and decision-making [26].

One of the most promising technologies for designing and implementing efficient smart sensors is the field programmable gate array (FPGA) because of their inherent parallelism, high performance, reconfigurability, and low cost [27]. Its parallelism feature allows several signal processing techniques to be implemented simultaneously on an FPGA. This is important for achieving a smart sensor with online monitoring and a fast response, which is mandatory for effective CT saturation detection [28,29]. Common FPGAs integrate a large number of resources, so implementing, in addition, a primary current reconstruction technique would be a straightforward task. Additionally, using an FPGA, the smart sensor can be easily reconfigured to obtain better results if it operates in extreme situations or conditions not considered during its original design.

Regarding the saturation detection algorithm, using the third central moment of the secondary current helps the smart sensor to distinguish the abrupt changes in the inrush current produced by CT saturation from those abrupt changes caused by power transformer saturation, which the second-order difference function is not capable of doing. Another disadvantage of derivative-based approaches to detect saturation is that their accuracy decreases with noisy signals [9], but in the proposed algorithm, this is also sorted out with the help of the third central moment. The higher-order statistics provide insight into signals, which is not always available at lower orders [30], such as variance or autocorrelation. Additionally, Gaussian-distributed signals have the characteristic of disappearing at higher orders [30,31]. Because so much noise is Gaussian distributed, the third-order central moment is proper with noisy signals. So, by combining that two time-domain features, a robust and powerful algorithm is obtained, with immunity against noise and harmonics, in addition to total independency on the type of CT saturation and type of transient current.

The saturation detection algorithm has been tested offline with simulated signals of transformer energizations and fault currents contaminated with Gaussian noise. Then, the smart sensor was physically implemented and tested in online conditions using synthetic simulated signals with the help of a hardware-in-the-loop (HIL) platform, in order to verify if it can fulfill the real-time and noise immunity requirements of an industrial relay. The effectiveness of the smart sensor has also been tested at different sampling rates and against the use of an anti-aliasing low-pass filter, which can smooth the signal, affecting CT saturation detection.

## 2. CT Saturation Detection Algorithm

### 2.1. CT Model and Saturation

**Figure 1:**
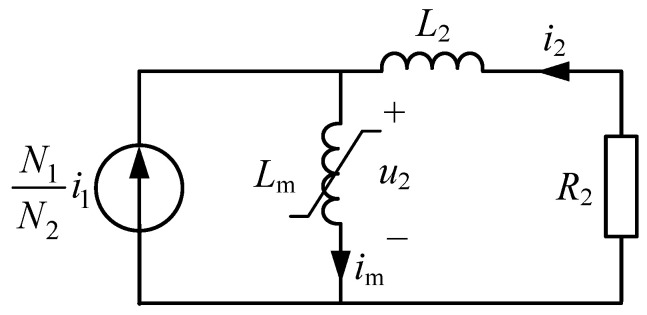
Simplified CT model.

Figure 1 shows a simplified equivalent circuit of a CT, suitable for transient analysis. It includes the nonlinear magnetizing inductance of the core, the resistance *R*_2_, and the inductance *L*_2_. In *R*_2_ are included the secondary winding resistance and the burden resistance, while *L*_2_ comprises the secondary leakage inductance and the burden inductance.

The circuit of Figure 1 is solved by writing Kirchhoff’s voltage law around the secondary (right) loop, as

(1) $$u_{2}-i_{2}R_{2}-L_{2}\frac{di_{2}}{dt}=0$$

where the induced voltage at the magnetizing inductance, *u*_2_, is given by

(2) $$u_{2}=\frac{d\phi }{dt}$$

and *i*_2_ is the current through the secondary winding, which using the nodal rule can be expressed as a function of the primary current, *i*_1_, and the magnetizing current, *i*_m_, as

(3) $$i_{2}=\frac{N_{1}}{N_{2}}i_{1}-i_{m}$$

where *N*_1_ and *N*_2_ are the primary (commonly equal to 1) and secondary winding turns, respectively.

The magnetizing behavior of the CT core is represented by the nonlinear inductance, *L*_m_, which has different values of inductance for different states of operation. It has a high value under normal conditions (it tends to behave as if it is similar to an open-circuit) and a low value (it tends to behave as if it is similar to a short-circuit) when the CT is saturated. The magnetizing current *i*_m_ is related to the flux by the saturation curve, a nonlinear function that characterizes the nonlinearity of *L*_m_. For the saturation detection algorithm developed in this section, it is not necessary to know the CT saturation curve in detail, because the detection is based only on the shape of the measured secondary current. However, it is necessary to know the information about the core flux and its behavior.

Substituting Equation (2) in Equation (1) and neglecting *L*_2_ because under application of modern numerical relays the burden can be considered fully resistive [9], the magnetic flux in the core is given by

(4) $$\phi =R_{2}\int i_{2}dt+\phi_{R}$$

where $\phi_{R}$ is the residual flux in the CT core. Equation (4) shows that the flux is proportional to the integration of the measured secondary current.

**Figure 2:**
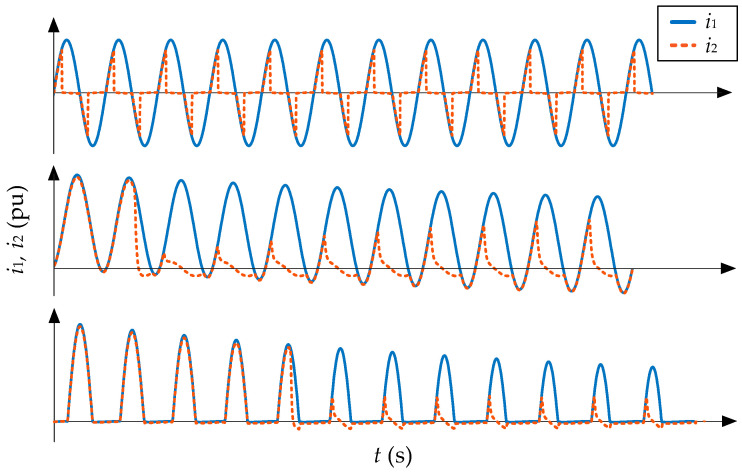
Typical waveforms of different saturated secondary currents (dotted lines) against primary currents (solid line): large symmetrical current (**top**), fault current (**center**), and inrush current (**bottom**).

As stated in the previous section, there are two types of CT saturation: symmetrical and asymmetrical. The first one happens when the primary current is too large, so the secondary current is chopped (dropped abruptly to zero) during saturation intervals twice every cycle (once during the positive half cycle and once during the negative half cycle). The main interest in this paper is asymmetrical saturation, which happens when the applied primary current has high levels of DC offset, as is the case of the inrush currents in power transformers. With this type of CT saturation, the secondary current is saturated only once at each cycle and every during the same half cycle (always positive or negative). Figure 2 shows typical waveforms of these two types of saturation and different types of currents.

It is important to highlight that even though the asymmetrical saturation is caused mainly by the DC offset, the magnitude current and other factors influence it. The time it takes for the CT to saturate, the duration of the saturation intervals, and the shape of the secondary current at the saturation intervals also depend on the impedance and type of connected burden, on the CT residual flux, and the *X*/*R* ratio of the analyzed system.

If there is residual flux with the opposite polarity of the current, more time is required for the CT to achieve saturation, while less time is required if they have the same polarity. The current magnitude also proportionally influences the time it takes for the CT to saturate: greater magnitude implies less time. The amount of time the CT is saturated is less at each cycle as the DC component decays, which is ruled by the *X*/*R* ratio of the power system in the case of a fault current, or by the transformer in the case of an inrush current. The inrush current magnitude is usually lower than fault current magnitude; however, its decaying time constant is larger.

Finally, the shape of the current during saturation intervals depends mainly on the burden. If it is purely resistive, the saturated current will drop abruptly to zero, but if the burden is also inductive, the saturated current has a slower decay. The development of saturation detection algorithms is essential because not all saturated waveforms are as evident as those shown in Figure 2, which have sharp edges and largely missed intervals. With low levels of saturation, detection is more complicated.

### 2.2. Time-Domain Features

The proposed smart sensor is based on the second-order difference function to detect abrupt current changes and, therefore, when the saturation starts at each cycle.

The second-order difference of the current *i*_2_ at the *n* instant, can be obtained as

(5) $$di_{2}\left(n\right)=i_{2}(n)-2i_{2}(n-1)+i_{2}(n-2)$$

as a function of the *n* current sample and the last two previous samples.

**Figure 3:**
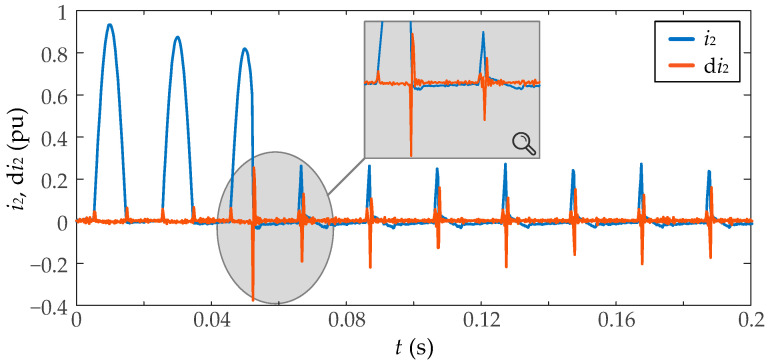
Second-order difference function (d*i*_2_) of a saturated inrush current (*i*_2_), and an enlargement of both signals during the first two saturation intervals.

As seen in Figure 3, the second-order difference function has peaks every time the measured current has a steep change, so the CT saturation can be detected. However, the regular changes in inrush current due to power transformer saturation also can be incorrectly detected as CT saturation inceptions. Moreover, it also presents lower peaks as a consequence of noise. Then, to improve the use of this function, the combined use of the third-order statistic central moment is suggested, which is one of the higher-order statistics.

A central moment is a statistic moment of a probability distribution of a random variable or discrete-time series about its mean; that is, it is the expected value of a specified integer power of the deviation of the random variable from the mean [30]. A higher-order moment relates to the spread and shape of the distribution.

**Figure 4:**
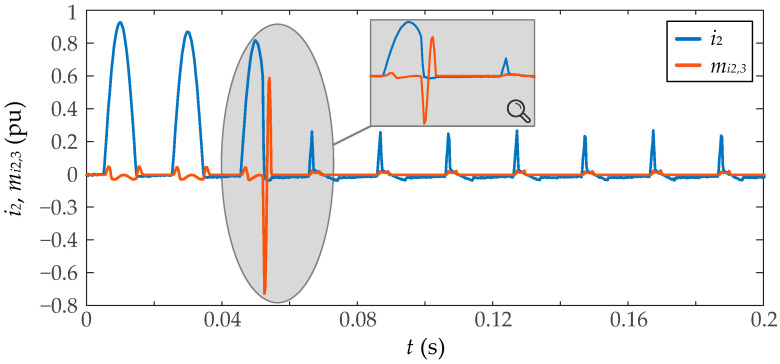
Moving third-order central moment (*m_i_*_2,3_) of a saturated inrush current (*i*_2_), and an enlargement of both signals during the first two saturation intervals.

The third central moment, *m*_3_, of a time-discrete series *x*(*k*) is defined as [30]

(6) $$m_{3}=\frac{1}{N}\sum_{k=1}^{N}{\left(x\left(k\right)-\bar{x}\right)}^{3}$$

where *N* is the number of samples and $\bar{x}$ is the time-series mean. This definition does not take into account the normalization around the standard deviation. This paper uses a sliding window along the measured current to obtain a *moving* version of the third central moment. If the window has a length of *L* samples, the moving third moment can be calculated as

(7) $$m_{i_{2},3}\left(n\right)=m_{3}\left[i_{2}(n),\;i_{2}(n-1),\;\ldots ,\;i_{2}(n-L+1)\right]$$

where it is considered an overlap of *L* − 1 samples between each adjacent window. Figure 4 shows an example of the moving third moment for an inrush current.

### 2.3. Start of Saturation

In order to detect a CT saturation inception due to a transient primary current, the following algorithm has to be accomplished:

1. Set an initial threshold value, *TH*, equal to 0.05 pu;
2. Set a third-moment threshold value, *m*_3*TH*_, equal to 0.003 pu;
3. Calculate in real time the two time-domain features (d*i*_2_ and *m_i_*_2,3_), with Equations (5) and (7), for the secondary CT current. To calculate *m_i_*_2,3_, it must be considered an overlap *L* equal to 10 samples;
4. Detect maximum or minimum local peaks in *m_i_*_2,3_ and compare them with the threshold value *TH*. If the absolute peak value is greater than the absolute value of existing *TH*, the latter will be updated with the peak value;
5. To detect the first CT saturation inception at *n* instant, it must be fulfilled that:
  - If *TH* is positive, d*i*_2_(*n*) must be negative with an absolute value greater than the threshold value;
  - If *TH* is negative, d*i*_2_(*n*) must be positive and greater than the absolute threshold value;

6. The *TH* value is updated with the third part of d*i*_2_(*n*) value, corresponding to the first CT saturation;
7. The subsequent CT saturation inceptions are detected if:
  - If *TH* is positive, d*i*_2_(*n*) must be positive and greater than the threshold. In addition, *m_i_*_2,3_(*n*) or *m_i_*_2,3_(*n* − 1) must be different from zero and with an absolute value greater than *m_3TH_*;
  - If *TH* is negative, d*i*_2_(*n*) must be negative and lower than the threshold value. Furthermore, *m_i_*_2,3_(*n*) or *m_i_*_2,3_(*n* − 1) must be different from zero and with an absolute value greater than *m_3TH_*.

**Figure 5:**
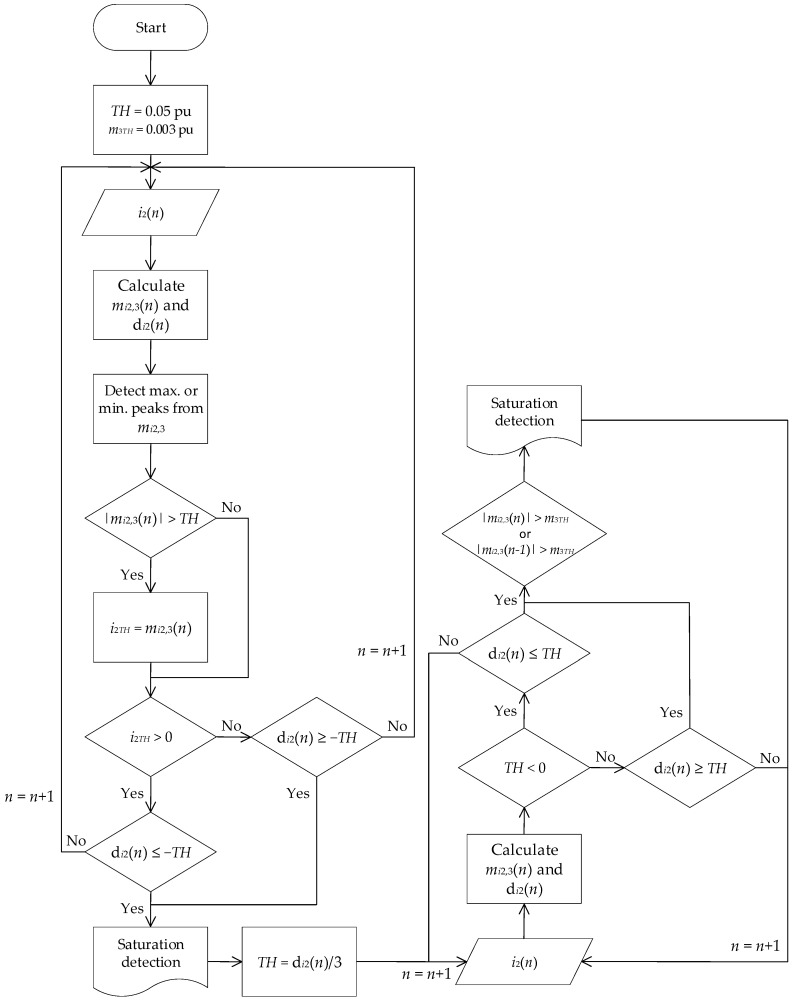
Flowchart of the CT saturation detection algorithm. (*TH*: threshold value, *m*_3TH_: third moment threshold value, *i*_2_: secondary CT current, *m_i_*_2,3_: moving third-order central moment, d*_i_*_2_: second-order difference function, *n*: sample number).

Figure 5 shows the flowchart that summarizes this CT saturation detection algorithm.

### 2.4. End of Saturation

It can be assumed that at the saturation instant, the CT core flux is the same as when the saturation ends. According to Equation (4), the CT flux is proportional to the integration of the secondary CT current. It is necessary to know the residual flux $\phi_{R}$ and *R*_2_ values to obtain the CT flux. Because $\phi_{R}$ only displaces the flux about the vertical axis and *R*_2_ scales the flux waveform, a *pseudo-flux* proportion to the actual flux can be obtained at the *n* time using the trapezoidal rule as

(8) $$\tilde{\phi }\left(n\right)=\tilde{\phi }\left(n-1\right)+\frac{i_{2}\left(n\right)+i_{2}\left(n-1\right)}{2}\left[t\left(n\right)-t\left(n-1\right)\right]$$

Therefore, the end of the saturation interval can be determined when the instantaneous *pseudoflux* magnitude falls below the magnitude corresponding to the saturation instant.

## 3. Smart Sensor

**Figure 6:**
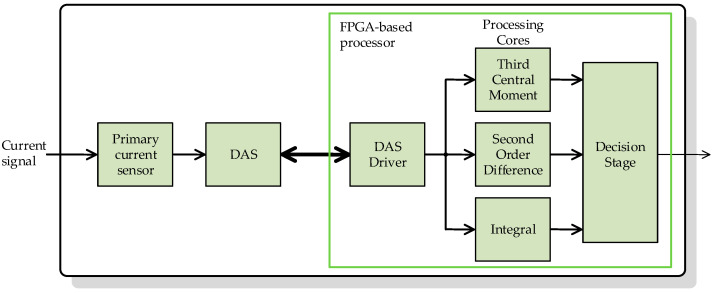
Architecture of the proposed smart sensor.

The block diagram of the general architecture of the proposed smart sensor is shown in Figure 6 The smart sensor is divided into three main stages: a primary sensor, a data acquisition system (DAS), and an FPGA-based processor.

The primary sensor stage consists of a current sensor (as a Hall Effect clamp meter) connected to the secondary CT side. As explained in the Section 1, the smart sensor has not been tested on fully real conditions, so the primary sensor has not been included on the prototype. The CT saturation detection algorithm has been tested using Simulink computer simulations, and the smart sensor prototype (FPGA-based processor) has been implemented in a dSPACE MicroLabBox platform (which incorporates a Xilinx FPGA) and tested with the help of a HIL (Typhoon platform), which provides in real time the *measured* CT secondary current signal.

The MicroLabBox incorporates analog to digital converters with a 16-bit resolution, a sampling frequency of 1 million samples per second (sps), and an input range from −10 V to +10 V. In this paper, the measured signal has been resampled internally in the DSPACE to reduce the sampling frequency to 4000 sps, which is in the range of the common sampling frequencies in digital relaying systems. The signal conditioning previous to the conversion includes a fully-differential isolation amplifier to obtain electrical isolation and a low-pass anti-aliasing filter, allowing the correct harmonic analysis.

The FPGA-based processor is the smart sensor’s final stage, responsible for the CT saturation detection, performed by the two time-domain features processing cores, an integrator core, and a decision stage. All these cores are described in detail in the following subsections. This processor delivers the saturation indicator signal, which can be sent to another device so that an optional communication interface can also be implemented in the FPGA. The FPGA-based processor also includes the necessary drivers for proper communication with the DAS and the finite state machine (FSM), which is necessary to handle the operation of all the processing cores.

### FPGA-Based Processor

The FPGA-based processor consists of two main stages. The first stage contains the two time-domain features processing cores and the integrator core, and the second stage decides whether there is saturation.

These processing cores are fully implemented on a single FPGA (Xilinx Kintex-7 XC7K325T), and the authors fully developed them under Very high speed integrated circuit Hardware Description Language (VHDL) and the standard libraries from IEEE. Commercially available processing cores and libraries have not been used.

**Figure 7:**
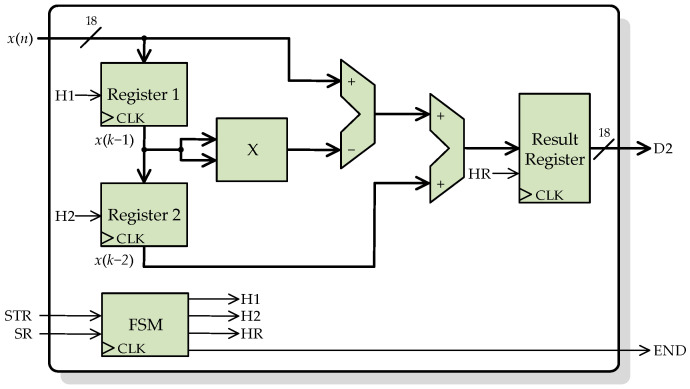
Architecture of the processing core for computing the second-order difference function.

Figure 7 shows the block diagram of the general architecture of the processing core for the second-order difference function, according to Equation (5). There are three input signals, *x*(*n*), STR, and SR, and two output signals, D2, and END. *x*(*n*) is the secondary CT current to be processed, a signal of 18-bit in a 2.16 fixed-point format. STR is a 1-bit indicator signal to start the calculation, and SR is a 1-bit signal to indicate to the processing core that a new *x*(*n*) sample is available to be read. D2 is the result of the processing core, an 18-bit signal with the same format as *x*(*n*). Finally, END is a 1-bit signal that indicates that a calculation has been finished and a new result is available to be read.

The processing core uses two parallel registers (Register 1 and Register 2) connected in cascade to store the last two input samples, *x*(*n* − 1) and *x*(*n* − 2). Each time a new sample is available at the input *x*(*n*), the two registers are enabled, so the last sample is stored, and the antepenultimate sample is discarded. There is another register to control the flow of the output result. The core also includes an FSM to control the enabling of registers and therefore the data flow. This FSM also handles the indicator signals (STR, SR, and END).

**Figure 8:**
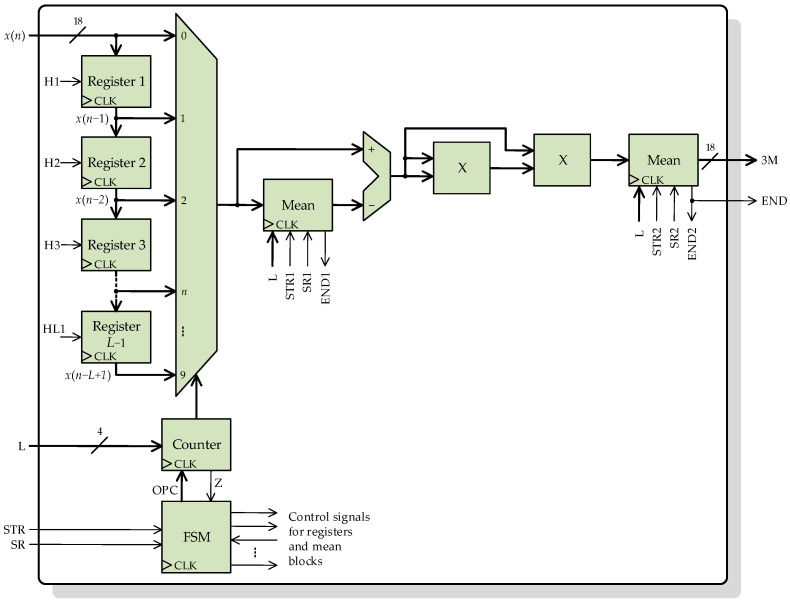
Architecture of the processing core for computing the moving third-order central moment.

Figure 8 depicts the general architecture of the second processing core for computing the moving third-order central moment according to Equations (6) and (7). This processing core has the same inputs and outputs as the previous processing core, plus a new 4-bit signal L, which indicates the length of the sliding window to the core. Again, *L* − 1 parallel registers connected in cascade to store the *L* − 1 last input samples. The input *x*(*n*) and the registers’ outputs, are connected through a multiplexor to a mean block. With the help of the multiplexor and a counter, the flow of current and past input samples can be controlled by the FSM. It is important to note that according to Equation (6), the mean of *L* input samples has to be subtracted from each sample, so the *L* samples must remain available until the mean calculation is finished. This is possible with the presented core design because the used FPGA has a base operating frequency (100 MHz) much larger than the sampling frequency. With two multipliers, the third power in Equation (6) is performed to finally obtain the mean again. The FSM handles all the indicator signals and the internal control signals for the mean blocks, registers, multiplexor, and counter.

**Figure 9:**
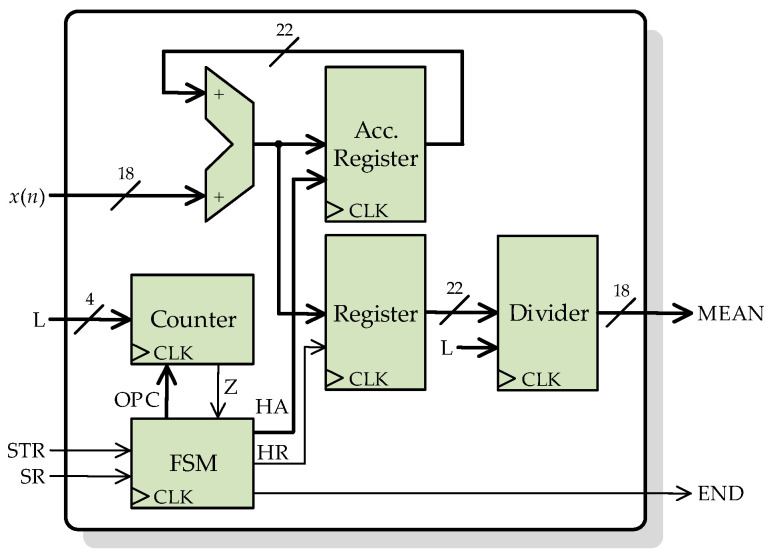
Architecture of the digital structure for computing the mean.

The mean structure, whose basic architecture is shown in Figure 9, is based on a digital structure known as accumulator. An accumulator is composed of an adder and two parallel registers. Both register inputs are connected to the adder output, whereas one register output is connected in feedback to one of the adder inputs. The function of this structure is to compute successive sums using only one adder. After the accumulator, a divider structure is used to divide the sum of all samples of the input signal *x*(*n*) between the number of samples *L*, obtaining the mean (18-bit MEAN signal). There is no division operator in the IEEE standard VHDL libraries, so it is necessary to design a digital structure for this purpose. The divider is based on a successive approximations register (SAR). This divider computes the division using a successive approximations approach. The SAR successively approximates the quotient value, comparing the quotient and divisor product, with the dividend until the product value is equal or very close to the dividend value.

**Figure 10:**
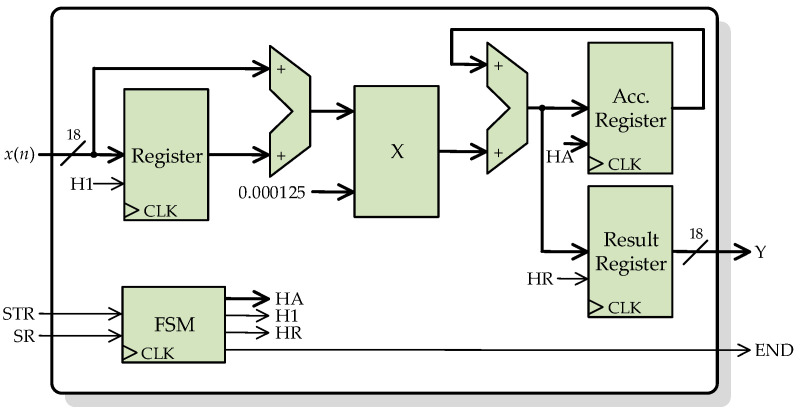
Architecture of the processing core for computing the integral.

Figure 10 shows the architecture of the last processing core for computing the integral of the secondary CT current, according to the trapezoidal rule. With a register at the input *x*(*n*), the processing core stores the previous sample, which is added to the current sample and then multiplied by a factor of 0.000125, which corresponds to half of the sampling period, (*t*(*n*) − *t*(*n* − 1))/2. Finally, successive sums are computed with an accumulator to obtain the cumulative integral at any time.

**Figure 11:**
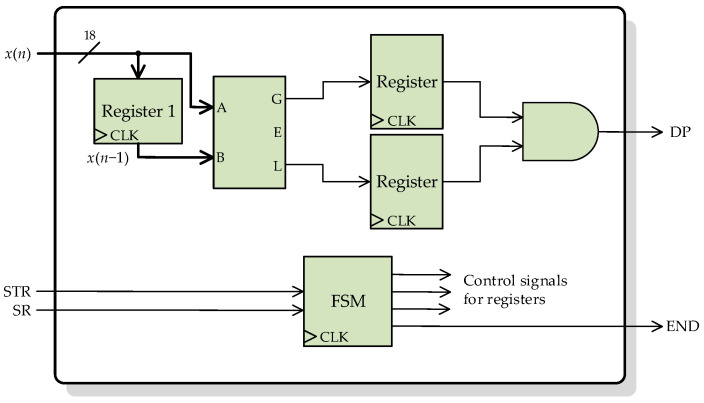
Architecture of the peak detector.

Finally, the decision stage is compounded by a simple peak detector and if-else decisions. Figure 11 shows the basic architecture of the peak detector, which is based on a comparator block.

Table 1 summarizes the resources usage of the FPGA and the processing time of each core in clock cycles, depending on the number of samples to be processed at each time (*L*) and the word length of the samples (i.e., *e* and *f*, which are the integer and fractional parts of each sample, respectively). For a period clock of 10 ns and a sampling frequency of 4000 sps, it is clear that the FPGA-based processor is fast enough to accomplish the real-time requirement.

## 4. Validation and Results

The proposed algorithm to detect CT saturation has been validated by simulations using MATLAB (algorithm implementation) and Simulink (CT model). It has been tested with fault short-circuit currents on a 120 kV network. The CT is rated 2000 A/5 A, 5 VA. The primary winding, which consists of a single turn passing through the CT core is connected in series with a shunt inductor rated 69.3 Mvar, 69.3 kV (120 kV/sqrt(3)), 1 kA rms. The secondary winding consisting of 400 turns is connected to a resistive burden. In the case of inrush currents, the algorithm has been tested using a 150 MVA transformer with a rated voltage of 289 kV.

**Figure 12:**
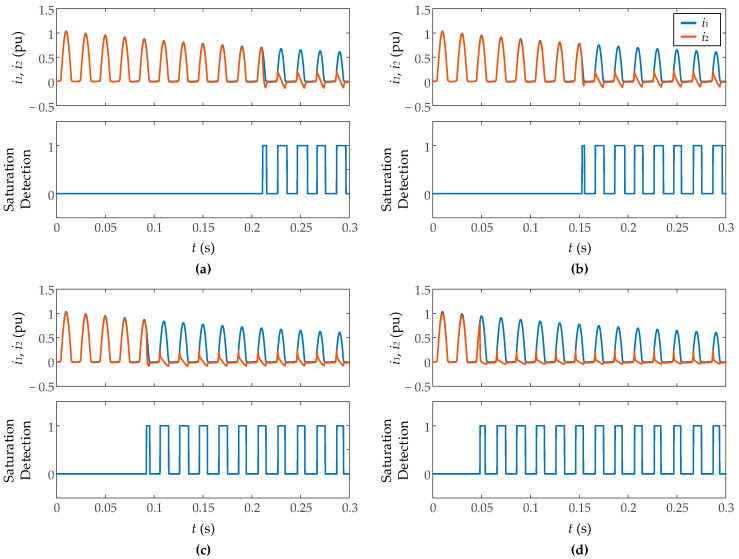
Performance of proposed algorithm on inrush currents for different resistive CT burdens: (**a**) 0.8 Ω, (**b**) 1 Ω, (**c**) 1.5 Ω, (**d**) 3 Ω.

**Figure 13:**
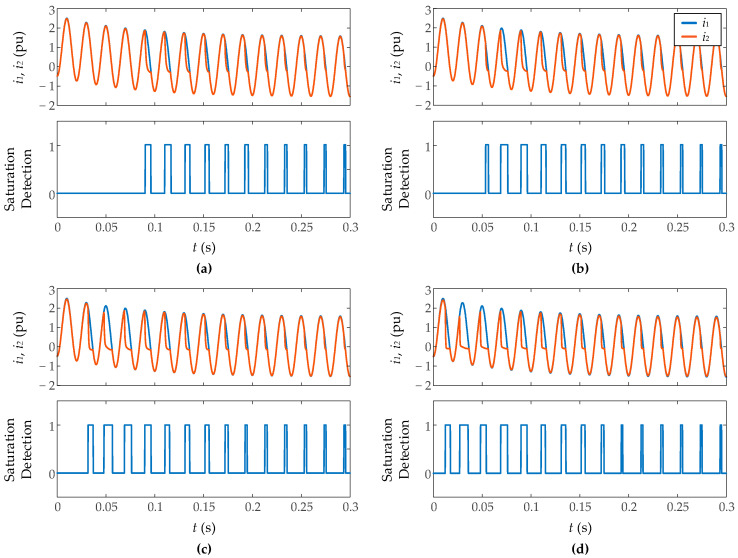
Performance of proposed algorithm on fault currents for different resistive CT burdens: (**a**) 0.8 Ω, (**b**) 1 Ω, (**c**) 1.5 Ω, (**d**) 3 Ω.

Figure 12 shows the results during inrush current measurement with different levels of resistance burden (0.8 Ω, 1 Ω, 1.5 Ω, and 3 Ω), inside the typical range of digital relays resistance. As explained before, more burden impedance implies a larger CT core flux, so the saturation is more severe with more resistance burden. In all cases, the algorithm detects with 100% efficiency the saturation without false positive detections. The saturation inception, in all cases, is detected just when the first sample of the measured secondary current does not coincide with that of the *ideal* secondary current without saturation. Regarding the end of saturation, the proposal fails at most one sampling period (0.25 ms), detecting in some cases the end of saturation a period after the event, but never before. This occurs with more frequency when the burden resistance is smaller. Figure 13 shows the results for fault currents with the same cases of resistance burden. The results are similar to the inrush currents, detecting even the light CT saturation on the last cycles.

**Figure 14:**
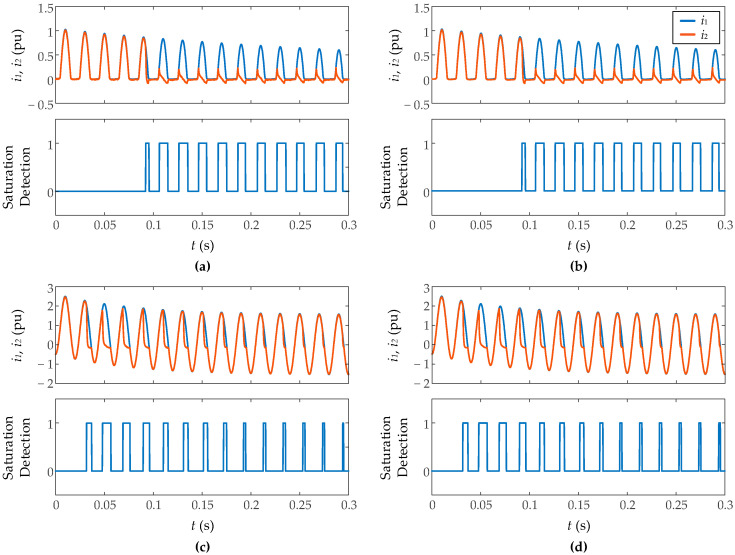
Performance of proposed algorithm against Gaussian noise. Signal to noise ratio: (**a**) 35 dB, (**b**) 50 dB, (**c**) 35 dB, (**d**) 50 dB.

**Figure 15:**
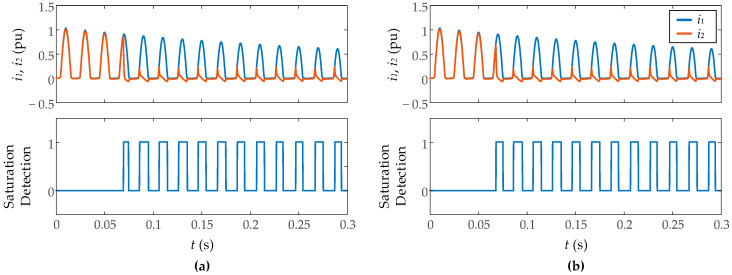
Performance of proposed algorithm against CT residual flux. (**a**) 0.2 pu, (**b**) 0.75 pu.

Figure 14 shows the results against inrush currents and fault currents measurement with different levels of Gaussian noise (signal-to-noise ratio of 35 dB and 50 dB) and a burden resistance of 1.5 Ω. It has been found that the algorithm ensures good results starting from a signal-to-noise ratio of 35 dB, which validates the immunity against noise of the algorithm. Figure 15 presents the results during inrush current measurement with different levels of CT residual flux (0.2 and 0.75 pu). More residual flux implies an earlier saturation, but not more severe saturation so the results are very similar in both cases without notable differences.

**Figure 16:**
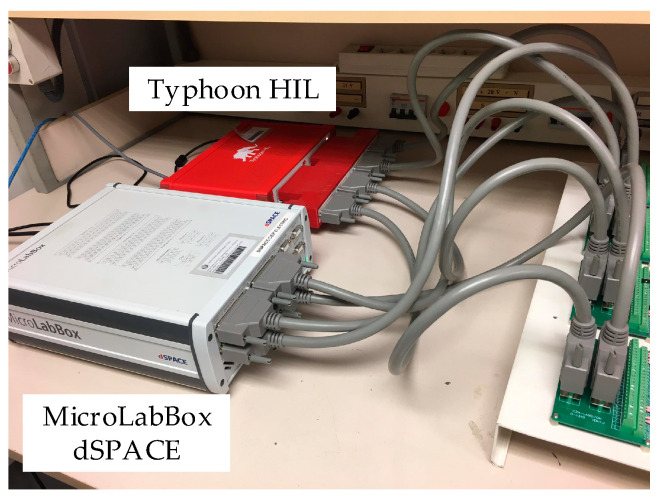
Experimental setup for real time test.

Finally, the smart sensor has been tested in real-time conditions. As explained, a hardware-in-the-loop platform (Typhoon HIL) has been used to emulate a power transformer energization (100 MVA, 289 kV), and the measurement with a 2000:5 CT (with a different saturation curve than the one used in the simulations), and the signals are sent to the smart sensor implemented in an FPGA in a MicroLabBox dSPACE. Figure 16 shows the experimental setup for these tests.

**Figure 17:**
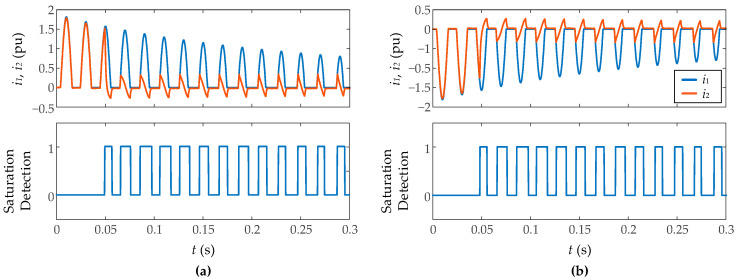
Performance of smart sensor during inrush current measurement in real time.

In Figure 17, the results for the measurement of two inrush currents with different polarity are shown. The saturation inceptions have been correctly detected with 100% efficiency. Regarding the end of saturation, the proposal fails at most one sampling period, detecting in some cases the end of saturation a period after the event.

**Figure 18:**
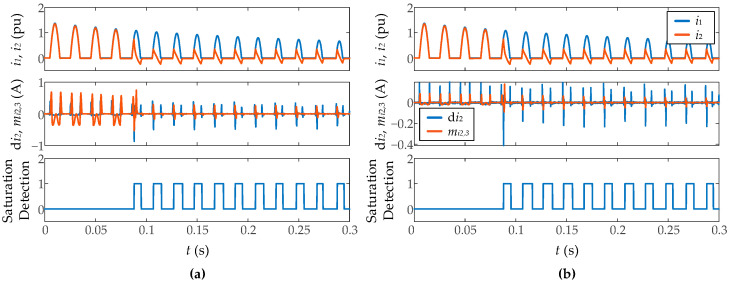
Performance of smart sensor during inrush current measurement in real time.

It has also been tested the influence of the sampling frequency (Figure 18). It has been found that higher sampling frequencies lead to a more accurate end-of-saturation detection. With sampling frequencies smaller than 4000 sps, the algorithm does not ensure good results because the threshold levels and sliding window length established in the Section 2 have to be changed. This is because at different sampling frequencies, the magnitudes of the two used time-domain features change, even for the same signal, as seen in Figure 18.

## 5. Conclusions

In this paper, a new methodology and its implementation into an FPGA-based smart sensor to detect the CT saturation mainly during inrush conditions is presented. The methodology and the smart sensor are tested and validated by changing next features for both simulation and real time scenarios: the burden resistance, the signal-to-noise ratio, the CT residual flux, and sampling frequency. Good results have been achieved in all cases, mainly to detect the initiation of CT saturation. Regarding the end of saturation detection, the methodology can be improved if the secondary winding inductance and the burden impedance are considered, in order to calculate an accurate core flux, instead of the *pseudoflux* used in this paper. For this, it would be necessary to consider that the FPGA-based processor should be reconfigured for each power system.

Attention must be paid to choosing the correct values for the thresholds and the length of the sliding window in calculating the moving third central moment, depending on the sampling frequency used.

Unlike most published CT saturation methodologies, the proposed algorithm shows effective results during inrush current measurement.

It has also been demonstrated the potential of an autonomous FPGA-based smart sensor with the integrated capability of signal processing and decision-making. The processing cores use very low FPGA resources, showing that this technology is well suited for designing and developing high-performance signal processing methods for smart sensors. One of the main advantages of this smart sensor is its simplicity and low computational cost with high-speed processing. It can also quickly adapt to operate online with any relay or measurement system.

## Figures and Tables

**Table 1:** Usage of FPGA resources.

Processing Core	Logic Elements	Registers	9-Bit Multipliers	Memory Bits	Clock Cycles
Second-order difference	480	56	2	0	2
Third central moment	1900	430	8	0	2*L* + 2(*e* + *f*) + 1
Integral	494	74	2	0	2
Decision stage	254	80	0	0	3

## Data Availability

Not applicable.
